# Immune checkpoint inhibitor associated lichenoid dermatitis mimicking erythema multiforme major with a germline variant in human leukocyte antigen-B15

**DOI:** 10.1016/j.jdcr.2024.11.035

**Published:** 2024-12-13

**Authors:** Sach Thakker, Alexis Antonucci, Amin Benyounes, Blair S. Allais

**Affiliations:** a Georgetown University School of Medicine, Washington, District of Columbia; b Virginia Commonwealth University School of Medicine, Richmond, Virginia; c Inova Schar Cancer Institute Melanoma and Skin Cancer Center, Fairfax, Virginia; d University of Virginia School of Medicine, Charlottesville, Virginia

**Keywords:** drug reaction, erythema multiforme major, oncodermatology, PD-1 inhibitor, pembrolizumab

## Introduction

Lichenoid dermatitis is a common reaction observed in up to 5% of patients treated with immunotherapy targeting programmed death-1 (PD-1) or programmed death-ligand 1 (PD-L1).^1^ Presentation varies from typical flat topped violaceous papules to inverse, hypertrophic and bullous variants, and can involve oral and anogenital mucosa.^2^ Onset is on average 12 weeks after medication initiation but has been reported after 1 to 266 days.^3^ Erythema multiforme major is a severe form of erythema multiforme (EM) characterized by widespread targetoid plaques and erosions on the skin and mucous membranes. It is frequently associated with infections, particularly mycoplasma and herpes simplex virus (HSV), or as a reaction to medications, including PD-1 inhibitors.^4^

The role of genetic predispositions in the development of drug-induced dermatoses is gaining attention, particularly concerning germline variants. Notably, specific human leukocyte antigen (HLA) alleles have been linked to increased susceptibility to adverse drug reactions. In the context of PD-1 inhibitors, the identification of certain HLA alleles in patients has been correlated with a higher likelihood of developing severe mucocutaneous reactions. This connection underscores the potential for genetic screening to predict adverse drug reactions and guide personalized treatment strategies.^5^

## Case report

**Fig 1:**
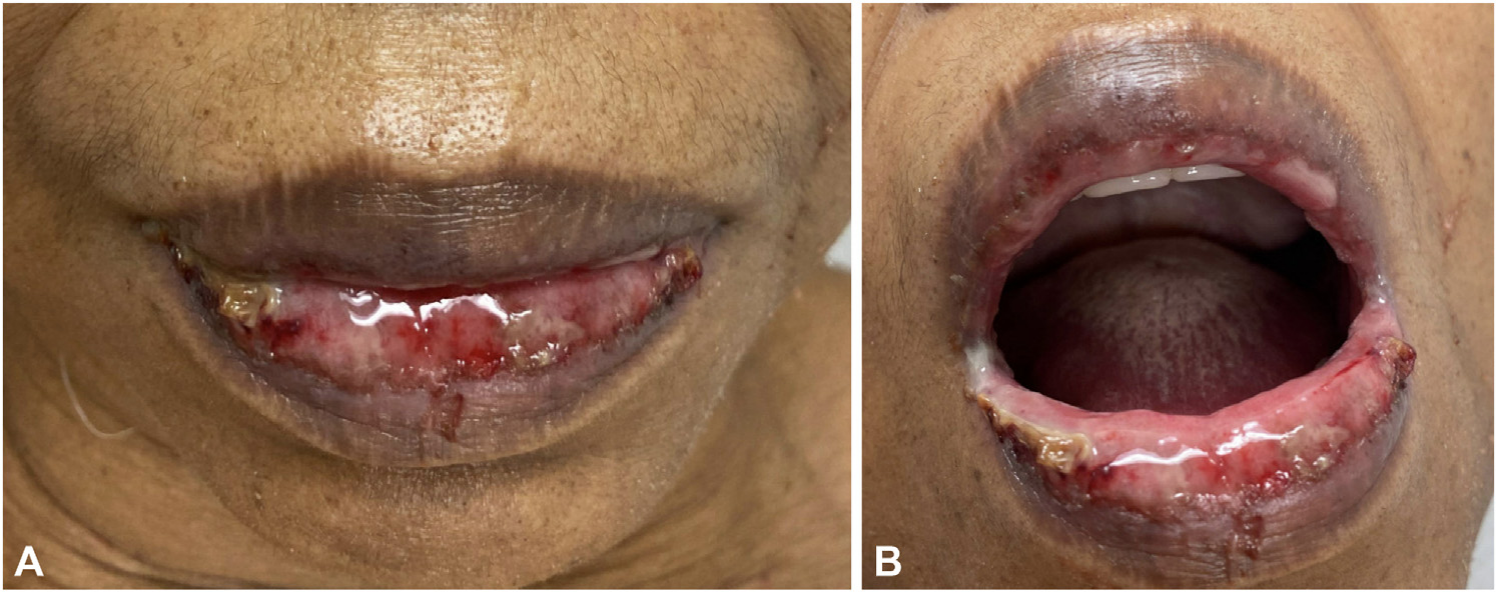
Upper and lower mucosal (**A** and **B**) lip with diffuse hemorrhagic oral erosions and crusting.

**Fig 2:**
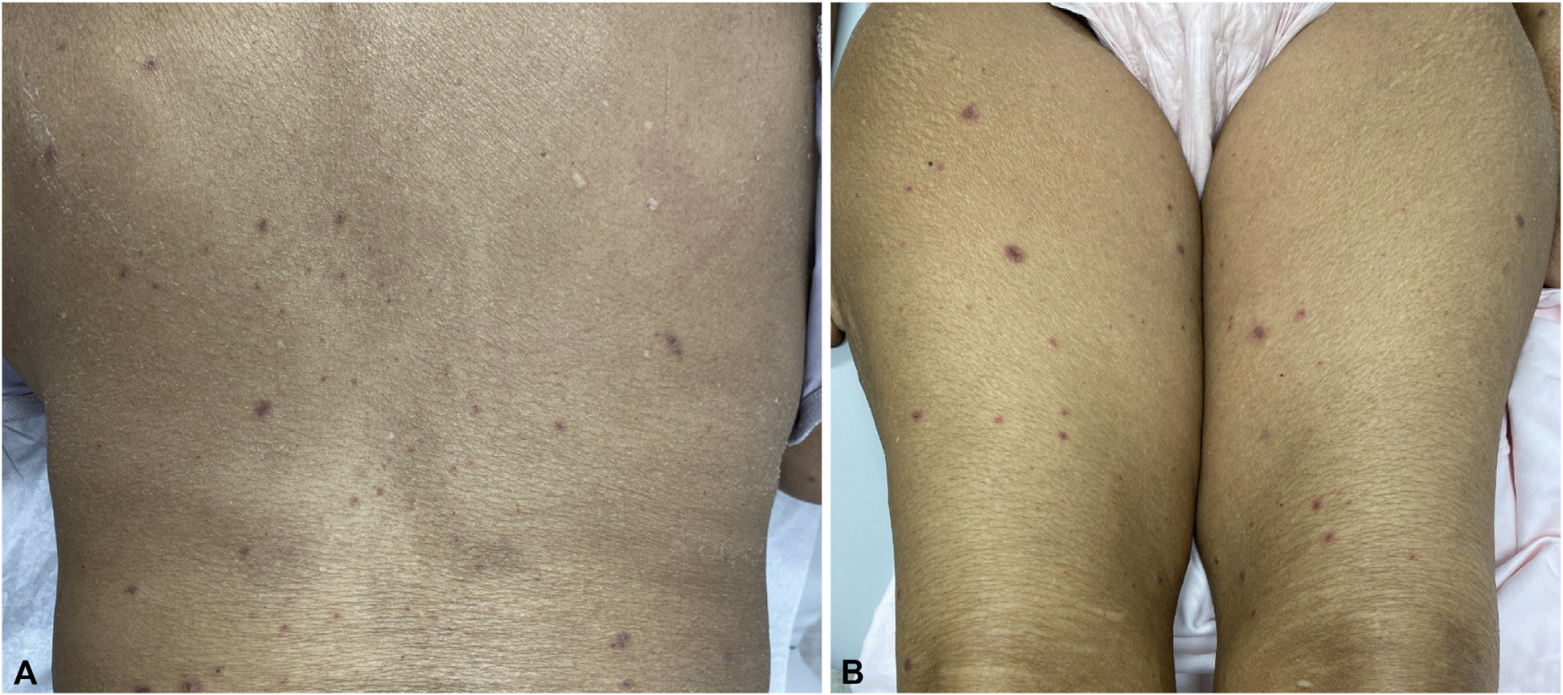
Scattered atypical targetoid papules to thin plaques seen on trunk (**A**) thighs (**B**) bilaterally.

**Fig 3:**
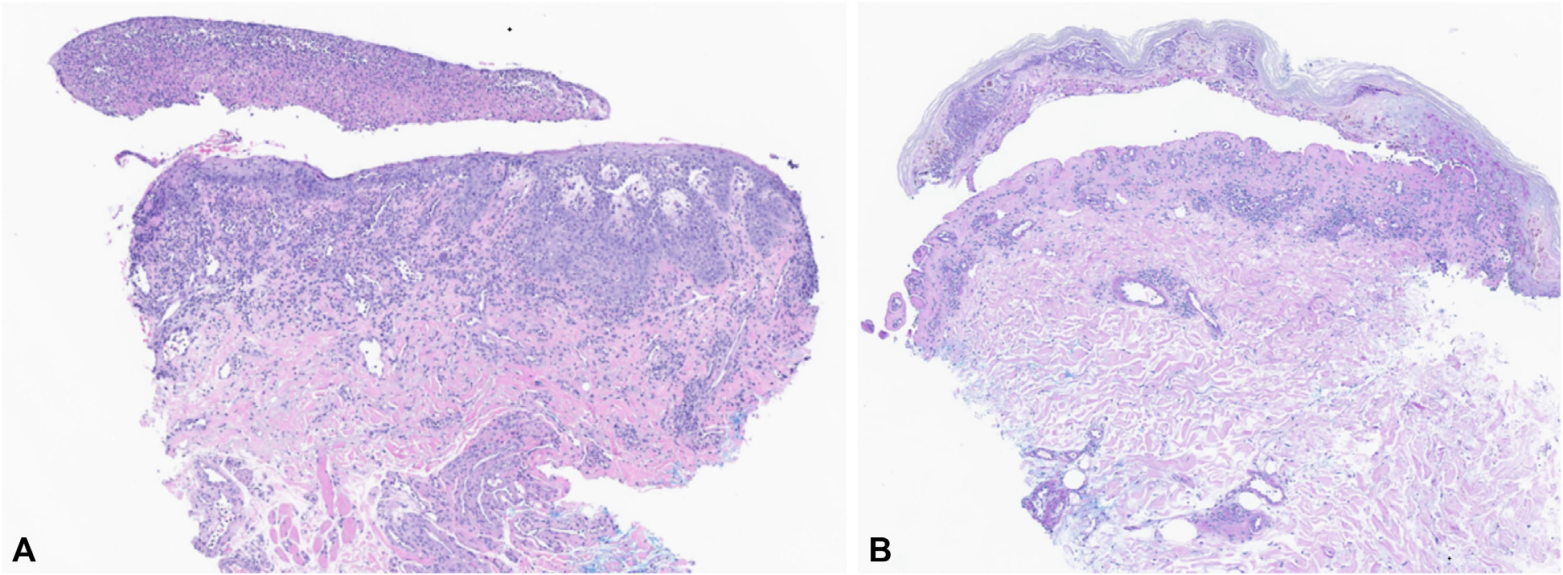
Punch biopsies of oral mucosa from the lower lip (**A**) and right thigh (**B**) consistent with epidermal necrosis with a sub epidermal split, superficial perivascular lymphocytic infiltrate, and vacuolization of the basal cell layer.

Our patient is a 72-year-old female with a history of tobacco use and stage IV squamous cell non-small cell lung cancer of the right lung with mediastinal adenopathy and bilateral pulmonary metastases. PD-L1 tumor proportion score was 50% at time of diagnosis (DAKO PD-L1 22C3). She initiated single agent pembrolizumab and presented to our clinic 17 days after her first infusion with pembrolizumab monotherapy reporting a 10-day history of worsening painful erosions in the mouth and a 3-day history of an asymptomatic rash on the trunk and extremities (Figs 1 and 2). Of note, she endorsed a foreign body sensation of the eyes, sore throat, lower leg weakness, and occasional chills but denied any fevers, skin pain, or dysuria. The patient denied starting any other medications during this time period. Physical examination was remarkable for conjunctival injection and yellow exudative discharge of the bilateral eyes as well as diffuse hemorrhagic oral erosions and scattered atypical two-toned targetoid papules to thin plaques on the trunk and extremities. Punch biopsies of the lower mucosal lip and right thigh revealed epidermal necrosis and apoptosis with a superficial perivascular lymphocytic infiltrate, basal layer vacuolization and a subepidermal split (Fig 3). HSV polymerase chain reaction swab of the oral mucosa was negative for HSV-1 and HSV-2.

**Fig 4:**
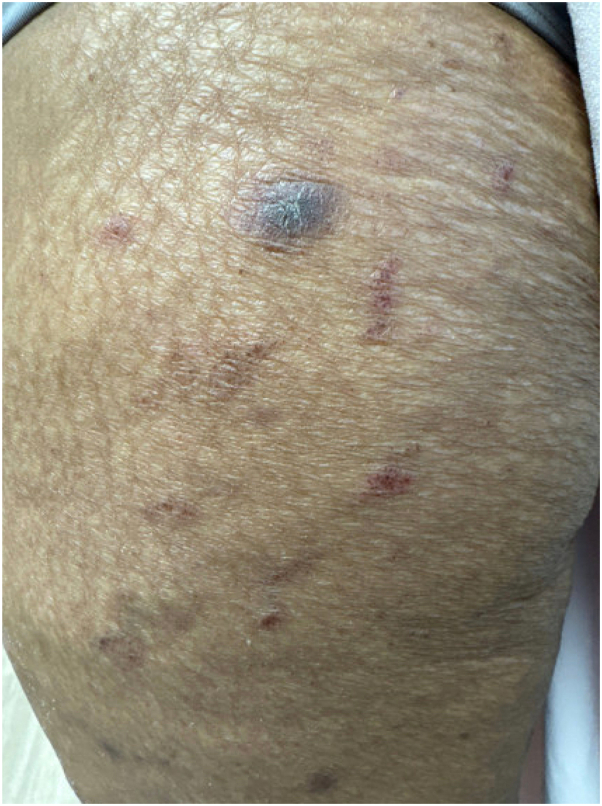
Purple thin plaque with overlying scale and scattered red to purple linear plaques and papules on the lower extremities at time of rash recurrence.

Our patient was referred for an urgent ophthalmologic examination which was consistent with anterior uveitis. She was successfully treated with a 60 mg oral prednisone taper, prednisolone 1% ophthalmic drops, clobetasol 0.05% ointment, triamcinolone 0.01% cream, and dexamethasone swish and spit rinses. Using a shared decision making model in light of her grade 3 cutaneous immune-related adverse event, she discontinued immunotherapy. Repeat imaging 8 weeks later demonstrated an interval decrease in size of target lesions in the right lung, subcarinal and paratracheal lymph nodes, consistent with a response to single dose immunotherapy. Six weeks after completing the prednisone taper, she developed recurrence of a scaly violaceous to erythematous rash on the extremities while not on anti-cancer therapy (Fig 4). There were no signs of uveitis. Repeat biopsy demonstrated a lichenoid interface dermatitis with eosinophils. The rash was successfully treated with twice daily triamcinolone 0.1% cream and cetirizine.

Interestingly, our patient later reported in follow-up that her sister had been treated with pembrolizumab and developed a similar mucocutaneous eruption shortly after starting. Broad panel genomic testing of the patient identified an allele in HLA B-15:16.

## Discussion

Upon initial presentation, the differential diagnosis for our patient included Immune Checkpoint Inhibitor (ICI) induced EM (specifically EM major given suspected concurrent cutaneous, ocular, and mucosal involvement and presence of atypical targetoid lesions), lichenoid dermatitis, early evolving Stevens-Johnson Syndrome/Toxic Epidermal Necrolysis, and Progressive Immunotherapy-Related Mucocutaneous Eruption, which presents with a milder disease course as compared to Stevens-Johnson Syndrome/Toxic Epidermal Necrolysis and involves a second drug trigger.^6^ While lichenoid dermatitis is generally not associated with ocular involvement, concurrent irAEs are common, and at least one study has demonstrated that patients receiving ICIs as therapy who developed colitis were also susceptible to episcleritis or uveitis.^7^ Given our patient’s recurrence of biopsy proven lichenoid dermatitis 12 weeks after ICI initiation, we suspect that our patient’s initial presentation was consistent with concurrent lichenoid dermatitis and anterior uveitis mimicking EM major. Recent research has also demonstrated increased keratinocyte apoptosis in immune-related lichen planus as compared to spontaneous lichen planus,^8^ which may explain our patient’s significant initial erosive mucocutaneous presentation. Interestingly, genes involved in phagosome signaling and macrophage activation are also upregulated in immune-related lichen planus as compared to spontaneous LP.

Our case also demonstrates the importance of germline variants in the development of cutaneous immune-related adverse events. Notably, a recent systematic review and meta-analysis looking at 322 patients who developed severe cutaneous adverse reactions following treatment with sulfamethoxazole/cotrimoxazole identified associations with HLA alleles, including the HLA-B∗15:02 allele.^9^ Testing for these alleles was performed by Tempus. In recent years, genomic testing in cancer patients has gained significant attention, with discussions highlighting its crucial role in guiding personalized treatment and improving cancer care strategies.^10^ As we continue to understand the role germline variants play in the development of cutaneous adverse reactions, our case builds on existing literature and highlights a unique presentation of a PD-1 inhibitor associated lichenoid dermatitis with an identified HLA B-15:16 allele. This case demonstrated the potential role of HLA-allele variants in the setting of severe cutaneous adverse reactions from immune checkpoint inhibition, especially in patients with refractory eruptions or a family history. This may inform future baseline testing prior to initiating immunotherapy.

## Conflicts of interest

None disclosed.
